# Chronic anticoagulation is not associated with a reduced risk of acute kidney injury in hospitalised Covid-19 patients

**DOI:** 10.1186/s12882-021-02436-5

**Published:** 2021-06-16

**Authors:** Kathrine Parker, Patrick Hamilton, Prasanna Hanumapura, Laveena Castelino, Michelle Murphy, Rachael Challiner, Jecko Thachil, Leonard Ebah

**Affiliations:** 1 Manchester Institute of Nephrology and Transplantation, Manchester University NHS Foundation Trust, Oxford Road, Manchester, M13 9WL UK; 2 Manchester Academic Health Sciences Centre (MAHSC), Citylabs 1.0, Nelson Street, Manchester, M13 9NQ UK; 3 Wellcome Centre for Cell-Matrix Research, Division of Cell Matrix Biology and Regenerative Medicine, School of Biological Sciences, Faculty of Biology Medicine and Health, The University of Manchester, Manchester, M13 9PL UK; 4 Department of Haematology, Manchester University NHS Foundation Trust, Oxford Road, Manchester, M13 9WL UK

**Keywords:** Acute kidney injury, Acute renal failure, COVID-19, Anticoagulation, Renal replacement therapy, SARS-CoV2

## Abstract

**Background:**

Coronavirus-19 (COVID-19) has been declared a global pandemic by the World Health Organisation. Severe disease typically presents with respiratory failure but Acute Kidney Injury (AKI) and a hypercoagulable state can also occur. Early reports suggest that thrombosis may be linked with AKI. We studied the development of AKI and outcomes of patients with COVID-19 taking chronic anticoagulation therapy.

**Methods:**

Electronic records were reviewed for all adult patients admitted to Manchester University Foundation Trust Hospitals between March 10 and April 302,020 with a diagnosis of COVID-19. Patients with end-stage kidney disease were excluded. AKI was classified as per KDIGO criteria.

**Results:**

Of the 1032 patients with COVID-19 studied,164 (15.9%) were taking anticoagulant therapy prior to admission. There were similar rates of AKI between those on anticoagulants and those not anticoagulated (23.8% versus 19.7%) with no difference in the severity of AKI or requirement of renal replacement therapy between groups (1.2% versus 3.5%). Risk factors for AKI included hypertension, pre-existing renal disease and male sex. There was a higher mortality in those taking anticoagulant therapy (40.2% versus 30%). Patients taking anticoagulants were less likely to be admitted to the Intensive Care Unit (8.5% versus 17.4%) and to receive mechanical ventilation (42.9% versus 78.1%).

**Conclusion:**

Patients on chronic anticoagulant therapy did not have a reduced incidence or severity of AKI suggesting that AKI is unlikely to be thrombotic in nature. Therapeutic anticoagulation is currently still under investigation in randomised controlled studies to determine whether it has a potential role in COVID-19 treatment.

**Supplementary Information:**

The online version contains supplementary material available at 10.1186/s12882-021-02436-5.

## Background

The World Health Organisation (WHO) declared the novel coronavirus 19 disease (COVID-19) a global pandemic in March 2020 [1]. The majority of patients with COVID-19 suffer mild symptoms and do not require hospital admission. More severe disease typically presents with dyspnoea and acute respiratory failure. In addition to respiratory issues, severe COVID-19 has been linked to cardiovascular complications such as myocardial infarction and acute stroke as well as acute kidney injury (AKI) [2, 3]. A hypercoagulable state is characteristic of severe COVID-19, with marked elevations of fibrinogen, factor VIII, and D-dimer [4, 5], and high rates of venous thromboembolism (including pulmonary embolism). Patients critically ill due to COVID-19 have been reported to have thrombotic event rates in the range of 20–30% in studies from the Netherlands, Italy and France, despite the use of venous thromboembolism (VTE) prophylaxis [6–8]. These are somewhat higher than seen in non-COVID critically ill patients where VTE rates are reported around 6% despite prophylaxis [7, 9]. The thrombotic process has been suggested to start prior to hospitalization, with Lodigiani et al. identifying 50% of VTE events within 24 h of admission [8].

Although original reports from Wuhan suggested rates of AKI were relatively low at around 5% [10] further analyses showed much higher rates of AKI associated with severe COVID-19, reported in 40–75% of intensive care unit (ICU) patients in Europe and the USA [11–13]. AKI is also considered a negative prognostic factor for survival [10, 14, 15]. Multiple mechanisms have been proposed for AKI including ischaemic acute tubular necrosis secondary to vascular collapse, endothelial damage, casts secondary to rhabdomyolysis and coagulopathy [16–18]. Post-mortem studies from China and New York support renal microvascular thrombosis as a potential cause of AKI [19–21].

There is the potential that the kidney may be a target for direct virus-mediated injury from SARS-CoV-2 via its ACE2 protein [16]. Direct viral microvascular injury exposes the subendothelial matrix containing tissue factor (TF) and collagen [22]. This contributes to activation of platelets and the coagulation cascade leading to fibrin formation and local thrombosis. The TF released during endothelial cell damage also leads to activation of inflammatory mediators such as C-Reactive protein (CRP) and Interleukin 6 (IL-6). Increased IL-6 levels are associated with increased plasma fibrinogen [20] and inflammatory mediator release results in increased TF expression further enhancing the hypercoagulable state [22, 23].

If AKI is related to COVID-19 thrombosis and the thrombotic process has begun prior to hospitalisation it may be conceived that patients on chronic anticoagulant therapy would have a reduced risk of developing AKI. With AKI associated with both poorer survival and ICU requirement for renal replacement therapy (RRT) [10–12, 17] chronic anticoagulation may also provide additional benefits. This study identified all COVID-19 positive patients admitted to Manchester University NHS Foundation trust, one of the largest hospitals trusts in the North West of England, who were receiving anticoagulant therapy at least 1 month prior to admission. The aim was to identify if chronic anticoagulant therapy had any effect on the incidence and severity of AKI to shed light on whether thrombosis may play a role in AKI aetiology in those with COVID-19.

## Methods

This study is a retrospective review of all hospital admissions with laboratory confirmed COVID 19 across a large NHS trust from 1st March 2020 to 30th April 2020 inclusive. All adult patients admitted to Manchester University NHS Foundation trust, aged > 18 years, were included if they had a positive polymerase chain reaction test of the nasopharyngeal or lower respiratory tract for COVID-19. All day cases and known haemodialysis patients were excluded.

### Setting

The setting for this study was in the leading provider of tertiary and specialist healthcare services in Greater Manchester, Manchester University NHS Foundation Trust (MFT) Hospitals. It comprises 10 hospitals/managed clinical services across six separate sites, providing a wide range of services from comprehensive local general hospital care through to highly specialised regional and national services.

Electronic hospital records were used to collect demographic and co-morbidity data. Patients were followed up for 30 days following COVID-19 diagnosis. Anticoagulant status on admission was determined using hospital records including letters, discharge summaries and General Practitioner (GP) records. Patients were included if they had been taking the anticoagulant for more than 1 month and this was confirmed manually with medication issues.

### Acute kidney injury

Serum creatinine values were used to identify AKI defined according to Kidney Disease Improving Global Outcomes (KDIGO) criteria [15] as follows: stage 1—increase in serum creatinine by 26.5micromol/L within 48 h or a 1.5 to 1.9 times increase in serum creatinine from baseline within 7 days; stage 2—2.9 times increase in serum creatinine within 7 days; stage 3—3 times or more increase in serum creatinine within 7 days or initiation of RRT. Although an electronic AKI detection and alerting system has already been in place within the trust since March 2016 (Think Kidneys/NPSA Safety Alert NHS/PSA/RE/2016/007), manual confirmation of each AKI case was still carried out, to ensure a valid baseline creatinine result was utilised. Community acquired-AKI was defined as AKI present within 24 h of hospital admission. All in-patients who had AKI from 7 days prior to COVID positive date and after the positive result date were included. In the period March 2020 to May 2020 routine screening for COVID-19 was not undertaken on all patients admitted to Manchester University NHS Foundation trust unless they presented with cough or respiratory symptoms. It may have been the case that some patients had asymptomatic COVID-19 on admission but not presented with symptoms for up to 7 days after hospitalisation [24]. Any patients who had developed AKI and recovered 7 days prior to the COVID positive date were excluded. Urine output criteria to define AKI were not utilised due to documentation being unreliable. Patients without a baseline creatinine were deemed to have AKI if they required renal replacement therapy as per KDIGO criteria or were deemed as clinical AKI based on manual review of consecutive creatinine results.

### Blood results

Baseline blood results were the results on day 0 (day of the first positive COVID sample) and if no blood tests on that day then the sample nearest to the day 0 result (no greater than 7 days earlier were used).

### Physiological observations

Patientrack™ by Alcidion is an electronic clinical observation system which is used to collect the observations at the bedside of patients across MFT. Observation data (temperature, blood pressure, mean arterial pressure (MAP), respiratory rate, oxygen saturations and early warning score (EWS)) for the patient cohort was collected and the set of observations closest to the COVID-19 positive date was considered.

All methods were carried out in accordance with relevant guidelines and regulations.

The primary endpoint was to identify if chronic anticoagulant therapy had any effect on the incidence and severity of AKI. Secondary endpoints included information on mortality, admission to critical care and requirement of mechanical ventilation.

### Statistical analysis

Categorical variables are presented as number and percentage. Normality was assessed using the Shapiro-Wilk normality test. Non-parametric continuous variables are presented throughout as the median and interquartile range. Univariable and multivariable logistic regression was used to assess factors predicting the development of AKI. The multivariable model is based on factors deemed to be clinically relevant such as demographics, and those factors which were found to be significant in the univariable logistic regression. Post-analysis goodness of fit was assessed using Akaike information criterion (AIC), c-statistic and the Hosmer-Lemeshow test. Survival analysis was carried out using a Cox proportional regression model. The multivariable model was adjusted for demographics and variables showing significance at univariable analysis. A *p*-value less than 0.05 was considered statistically significant throughout and all analysis was carried out using R version 3.6.122.

## Results

### Demographics

From March 1, 2020 to April 30, 2020, a total of 11,721 patients were admitted to MFT hospitals with 1166 of these having a diagnosis of COVID-19. After excluding the 35 dialysis patients and 99 day cases, there were a total of 1032 COVID-19 positive patients included in the analysis. Of these there were 164 patients (15.9%) taking anticoagulants for a minimum of 1 month prior to admission. Overall the anticoagulated patients were significantly older 80 versus 68 years (*p* < 0.001), had a higher Charlson Comorbidity Index (CCI) 2 versus 1 (*p* < 0.001) and were more likely to have hypertension (HTN) (*p* = 0.042), renal impairment (*p* = 0.009), cancer (*p* = 0.004) and heart failure (*p* < 0.001) (Table 1). Patients taking anticoagulants were more likely to be of white race, 139 (84.8%) versus 586 (67.6%) of those not anticoagulated (*p* < 0.001).

**Table 1:** Demographics table for all patients and then stratified into whether they were taking anticoagulation at diagnosis of COVID-19

		Overall	Anticoagulation		p
			No	Yes	
N		1032	868	164	
Age		71 (56–83)	68 (54–82)	80 (70–85)	< 0.001
Sex	*Male*	569 (55.1)	485 (55.9)	84 (51.2)	0.311
Ethnicity	*White*	725 (70.3)	586 (67.6)	139 (84.8)	< 0.001
	*Asian*	116 (11.3)	106 (12.2)	10 (6.1)	
	*Black*	94 (9.1)	88 (10.1)	6 (3.7)	
	*Mixed*	14 (1.4)	14 (1.6)	0 (0.0)	
	*Other*	28 (2.7)	28 (3.2)	0 (0.0)	
	*Unknown*	54 (5.2)	45 (5.2)	9 (5.5)	
CCI Score		1 (0–2)	1 (0–2)	2 (1–3)	< 0.001
Myocardial Infarction		83 (8.9)	61 (7.8)	22 (14.3)	0.015
Congestive Heart Failure		129 (13.8)	77 (9.9)	52 (33.8)	< 0.001
Peripheral Vascular Disease		64 (6.9)	50 (6.4)	14 (9.1)	0.304
Cerebrovascular disease		45 (4.8)	38 (4.9)	7 (4.5)	1.000
Dementia		157 (16.8)	128 (16.4)	29 (18.8)	0.538
COPD		259 (27.7)	212 (27.2)	47 (30.5)	0.455
Rheumatoid Disease		23 (2.5)	16 (2.1)	7 (4.5)	0.123
Peptic Ulcer Disease		9 (1.0)	8 (1.0)	1 (0.6)	1.000
Mild Liver Disease		30 (3.2)	30 (3.8)	0 (0.0)	0.026
Diabetes without complications		237 (25.4)	191 (24.5)	46 (29.9)	0.193
Diabetes with complications		36 (3.9)	29 (3.7)	7 (4.5)	0.796
Hemi or Paraplegia		17 (1.8)	12 (1.5)	5 (3.2)	0.263
Renal Disease		144 (15.4)	109 (14.0)	35 (22.7)	0.009
Mod/Severe Liver Disease		12 (1.3)	12 (1.5)	0 (0.0)	0.247
Cancer		72 (7.7)	51 (6.5)	21 (13.6)	0.004
Metastatic cancer		24 (2.6)	14 (1.8)	10 (6.5)	0.002
AIDS		1 (0.1)	1 (0.1)	0 (0.0)	1.000
Hypertension		457 (44.3)	372 (42.9)	85 (51.8)	0.042

All results given in n (%) and median (IQR). Comorbidities based on ICD10 codes. *CCI score* Charlson Co-morbid Index score, *COPD* Chronic obstructive pulmonary disease. Renal disease defined as Charlson Co-morbid Index (renal transplant, uraemia or serum creatinine >265micromol/)

### Anticoagulant therapy

The most common anticoagulant therapy was the Direct Acting Oral Anticoagulants (DOACs), accounting for 115 (70.1%) prescriptions. Atrial fibrillation (AF) was the most frequent indication in 119 patients (72.5%), Table 2. For those taking Vitamin K antagonists (VKAs), the mean International normalised ratio (INR) on admission was 4.0 (SD 3.21). All patients admitted to the ICU on anticoagulants were switched to low molecular weight heparin (LMWH) therapy. At discharge, five patients previously on warfarin were switched to either a DOAC or LMWH.

**Table 2:** Anticoagulant on admission and indication

Anticoagulant (%)	
*DOAC*	115 (70.1)
*VKA*	44 (26.8)
*LMWH*	3 (1.8)
*Fondaparinux*	2 (1.2)
Indication (%)	
*AF*	119 (72.5)
*VTE*	41 (25)
*Metallic heart valve*	2 (1.2)
*LV thrombus*	2(1.2)

*VKA* vitamin K antagonist, *DOAC* Direct acting oral anticoagulant, *LMWH* Low molecular weight heparin, *AF* Atrial Fibrillation, *VTE* venous thromboembolism, *LV* left ventricular

### Acute kidney injury

AKI was seen in 39 patients (23.8%) who were on anticoagulation which was higher than those not taking anticoagulants 171 (19.7%) but this difference was not statistically significant (*p* = 0.278), Table 3. There was no difference in severity of AKI between the groups with a similar proportion of those who developed AKI experiencing stage 3 AKI; 19 (48.7%) of those on anticoagulation and 73 (42.7%) of those not (*p* = 0.783), Table 4. Fifty-nine percent of patients on anticoagulants had a community acquired AKI as did 59% of those not anticoagulated. The median time to development of AKI from COVID positive PCR result was 0 days (IQR − 2.5-2.5) in the anticoagulated group and − 1 days (IQR − 1-1) in the non-anticoagulated cohort. Renal replacement therapy requirement was not different between groups with 2 (1.2%) patients on anticoagulants versus 30 (3.5%) (*p* = 0.204). Seventeen patients were deemed to have AKI criteria without a baseline creatinine available, eight of them required renal replacement therapy as per KDIGO criteria and the remaining nine were deemed to have clinical AKI based on consecutive review of creatinine results.

**Table 3:** Outcomes of all patients and stratified for those who were taking anticoagulation or not anticoagulated

	Overall	Anticoagulation		*p*-value
		No	Yes	
n	1032	868	164	
Died (%)	326 (31.6)	260 (30.0)	66 (40.2)	0.012
30-day mortality (%)	278 (26.9)	221 (25.5)	57 (34.8)	0.018
Readmit within 30 days (%)	86 (8.3)	69 (7.9)	17 (10.4)	0.383
Acute Kidney Injury	210 (20.3)	171 (19.7)	39 (23.8)	0.278
RRT (%)	32 (3.1)	30 (3.5)	2 (1.2)	0.204
Went to Critical care (%)	165 (16.0)	151 (17.4)	14 (8.5)	0.006
Died in Critical care (%)	59 (35.8)	50 (33.1)	9 (64.3)	0.042
Ventilation (%)	124 (75.2)	118 (78.1)	6 (42.9)	0.009

Values reported as n (%). RRT – renal replacement therapy

**Table 4:** Outcomes for patients with AKI stratified depending on whether they took anticoagulation or not

			Overall-AKI	Not anticoagulated-AKI	Anticoagulated-AKI	*p*-value
n			210	171	39	
Died (%)			110 (52.4)	91 (53.2)	19 (48.7)	0.741
30-day mortality (%)			97 (46.2)	80 (46.8)	17 (43.6)	0.855
Readmit within 30 days (%)			13 (6.2)	10 (5.8)	3 (7.7)	0.95
Time to AKI from admission, days			0.00 (0.00–4.00)	0 (0.00–4.00)	1.00 (0.00–4.00)	0.966
Time to AKI from COVID diagnosis, days			−1.00 (1.00–2.00)	−1.00 (− 1.00–1.00)	0 (−2.50–2.50)	0.977
Initial AKI stage		*1*	123 (58.6)	99 (57.9)	24 (61.5)	0.380
		*2*	37 (17.6)	33 (19.3)	4 (10.3)	
		*3*	50 (23.8)	39 (22.8)	11 (28.2)	
Peak AKI stage		*1*	75 (35.7)	62 (36.3)	13 (33.3)	0.783
		*2*	43 (20.5)	36 (21.1)	7 (17.9)	
		*3*	92 (43.8)	73 (42.7)	19 (48.7)	
Day zero *Sodium*		*mmol/L*	138.00 (133.00–142.00)	138.00 (133.00–142.00)	138.00 (134.50–142.50)	0.758
Peak	*Urea*	*mmol/L*	18.40 (12.80–27.30)	18.25 (12.43–26.98)	18.60 (14.10–29.05)	0.591
	*Creatinine*	*μmol/L*	189.00 (136.00–305.00)	187.00 (135.25–311.50)	191.00 (143.50–260.50)	0.831
	*CRP*	*nmol/L*	208.00 (107.00–312.00)	231.00 (110.25–317.75)	169.00 (94.50–218.50)	0.054
Minimum	*Haemoglobin*	*g/L*	96.00 (76.00–117.00)	96.00 (74.25–116.75)	97.00 (86.00–124.50)	0.36
	*Lymphocytes*	*10*^*9*^*/L*	0.64 (0.41–0.89)	0.64 (0.41–0.95)	0.63 (0.47–0.86)	0.941
Went to critical care			73 (34.8)	65 (38.0)	8 (20.5)	0.06
Died in critical care (%)			38 (52.1)	33 (50.8)	5 (62.5)	0.801
Ventilation (%)			63 (86.3)	59 (90.8)	4 (50.0)	0.009

Values reported as n (%) or median (IQR 25th–75th centile)

Anticoagulation was not found to be a factor associated with AKI in multivariate analysis (OR 1.02 (0.60–1.68, *p* = 0.952)). Factors that were linked with AKI were pre-existing renal disease (OR 2.31 (1.42–3.72, *p* = 0.001)), HTN OR 1.61 (1.07–2.46, *p* = 0.024) and male sex (OR 1.65 (1.11–2.49, *p* = 0.015)), Table 5.

**Table 5:** Logistic regression for the development of AKI

Dependent: AKI		OR (multivariable)
Age		1.00 (0.99–1.02, *p* = 0.794)
Sex	*M*	1.65 (1.11–2.49, p = 0.015)
Ethnicity	*Asian*	0.69 (0.31–1.42, *p* = 0.335)
	*Black*	1.33 (0.70–2.45, *p* = 0.362)
	*Mixed*	0.47 (0.02–3.15, *p* = 0.512)
	*Other*	0.33 (0.02–1.76, *p* = 0.294)
	*Unknown*	1.14 (0.42–2.76, *p* = 0.775)
Anticoagulation		1.02 (0.60–1.68, p = 0.952)
Hypertension		1.61 (1.07–2.46, p = 0.024)
Diabetes with complications		2.27 (0.93–5.40, *p* = 0.065)
Renal disease		2.31 (1.42–3.72, p = 0.001)
Cancer		1.57 (0.77–3.11, *p* = 0.203)
Metastatic cancer		1.45 (0.44–4.31, *p* = 0.522)
Day zero^a^	*Sodium*	1.04 (1.01–1.07, p = 0.015)
	*CRP*	1.00 (1.00–1.01, *p* = 0.003)
EWS		1.11 (1.00–1.23, *p* = 0.046)

Number in dataframe = 1032, Number in model = 785, Missing = 247, AIC = 709.2, C-statistic = 0.708, H&L = Chi-sq(8) 4.76 (*p* = 0.783)

Multivariable models adjusted by demographics and variables showing significance at univariable analysis (Univariate analysis is found in Supplementary Table 1)

### Survival

Overall 326 (31.6%) patients with a diagnosis of COVID-19 died following hospital admission, 36 of these deaths occurred following admission in the community. Mortality was higher in the anticoagulant therapy cohort with 66 (40.2%) of patients dying compared to 260 (30%; *p* = 0.012). The 30 day mortality was also higher in those taking anticoagulants 34.8% versus those not previously taking them 25.5% (*p* = 0.018), Table 3. If looking at mortality specifically in patients with AKI there was no difference between those on anticoagulation and those not anticoagulated, *p* = 0.741 Table 4.

Compared to those not taking anticoagulants significantly fewer patients on anticoagulants went to ICU 14 (8.5%) versus 151 (17.4%) (*p* = 0.006), Table 3. Of those that were admitted to ICU, a much smaller proportion of anticoagulated patients received mechanical ventilation (*p* = 0.009). Patients on anticoagulants who were admitted to the ITU had a higher mortality than those who weren’t previously anticoagulated (*p* = 0.042).

Across the whole study population, a multivariable cox proportional hazards regression did not find anticoagulation to have any impact upon overall survival HR 0.84 (CI 0.63–1.12, *p* = 0.283), supplementary Figure 1, and in univariable analysis it was not an independent factor for mortality HR 1.27 (CI 0.97–1.67; *p* = 0.081), supplementary Table 2. Factors from this analysis that were associated with mortality included AKI, age, diabetes, cancer and heart failure, supplementary Figure 1.

## Discussion

This retrospective, single-centre study found that pre-existing therapeutic anticoagulation had no impact on the risk of developing AKI or reducing its severity in hospitalised patients with COVID-19. AKI was likely to be present on admission. This is the first study we are aware of to examine the effects of chronic anticoagulation on COVID-19 related AKI.

The findings of this study would suggest that renal microvascular thrombosis is unlikely to play a significant role in COVID-19 related AKI. Early histopathological analysis from Wuhan, China identified segmental fibrin thrombi in glomerular loops associated with endothelial injury and this was proposed as a contributing factor to COVID-19 related AKI [19]. However, in this analysis, not all patients displayed signs of kidney injury evidenced by raised creatinine. Further autopsy series including a report from New York also identified platelet rich fibrin microthrombi in peritubular capillaries and venules in kidney tissues from all seven patients studied [20, 21, 25]. There have also been suggestions that the intricate link between the immune system and coagulation pathways with microvascular endothelial injury and hypercoagulability may exacerbate the risk of developing acute tubular necrosis (ATN) [16]. A group looking at 12 renal biopsies with some degree of ATN showed no evidence of thrombotic microangiopathy or vascular/capillary thrombi [26]. As more biopsy studies have been published various pathologies have been seen including collapsing glomerulopathy [27] and acute tubular injury (ATI) which is the predominant finding in numerous case series [19, 26, 28, 29]. ATI is likely multifactorial due to sepsis, hypoxia, multiorgan complications and rhabdomyolysis [29, 30]. The kidney has also been identified as being a target for SARS-CoV-2 via its ACE2 protein expressed on cells of the proximal tubule supporting the potential for direct virus-mediated injury [16]. Standard AKI risk factors are still implicated [16, 30, 31] and we found this in our population with significantly more patients developing AKI that had pre-existing renal disease, hypertension, diabetes and peripheral vascular disease which was also seen in another publication [17]. Consideration of these risk factors need to be undertaken to identify those patients susceptible to AKI early in the disease course to try and minimise the risk of developing AKI or its progression [30].

Stage 3 AKI was seen in 12% of all patients taking anticoagulation and this finding is important to highlight the need to review the suitability of the anticoagulant depending on the patient’s condition [30]. Russo et al. found that COVID-19 patients with AKI had a higher risk of death from bleeding compared to the rest of the study population and postulated this may have been related to an overdose of anticoagulants [32]. In patients with COVID-19 and AKI stage 3, LMWH dosed as per local protocols for renal impairment with anti-Xa monitoring should be preferred due to the DOACs’ reliance on renal excretion and the variability of INR seen with vitamin K antagonists (VKAs) [33, 34]. Supratherapeutic INRs were seen in our patients at admission similar to a study from Kings College, London which also noted high INRs (INR > 8) mostly relating to the antimicrobial therapy used to treat COVID-19 pneumonia [35]. Patients chronically anticoagulated have been recommended to switch to DOACs, if appropriate, during the pandemic with less monitoring required than VKAs and this was seen in a proportion of our patients at discharge [36]. However a recent Medicines Health Regulatory Authority (MHRA) alert has highlighted an increase in reported major bleeding episodes with DOACs [37]. Due to potential interactions with the COVID-19 trial medication lopinavir/ritonavir [38] or macrolide antimicrobial therapy used for pneumonia, along with high rates of AKI associated with COVID-19 extra vigilance is required when using these drugs to ensure the dose is appropriate and any additional therapies are suitable in combination.

We found that significantly fewer patients taking anticoagulant therapy were admitted to the ICU and they were less likely to receive mechanical ventilation than those not anticoagulated. Our findings could be explained by agreed limitations of care in the anticoagulated population due to pre-existing co-morbidities, including metastatic cancer, and advanced age. Both of these are also likely to impact on ICU survival. All of the anticoagulated patients who became critically ill and were admitted to ICU were switched to therapeutic LMWH [39]. Additional benefits of LMWH in severe COVID-19 disease have been described with a systematic review concluding that heparin can decrease levels of inflammatory biomarkers [40]. Numerous mechanisms of how heparins do this are described in the literature to include binding to and neutralisation of cytokines, interference with leukocyte trafficking, reducing viral cellular entry, and neutralisation of extracellular cytotoxic histones which can cause endothelial damage [41].

The patients chronically anticoagulated had a higher co-morbid burden with an increased prevalence of heart failure, renal disease and hypertension compared to those not taking anticoagulation on admission (*p* < 0.05). Anticoagulated individuals were also of advanced age (80 vs 68 years) which makes it difficult to draw conclusions on overall survival between the groups. These are known risk factors for COVID-19 related mortality [42]. Recently a study carried out across the UK by the International Severe Acute Respiratory and emerging Infections Consortium Coronavirus Clinical Characterisation Consortium (ISARIC-4C) developed a risk stratification score for COVID-19 mortality where increasing age, multiple co-morbidities and urea > 14 mmol/L are some of the major contributing factors to mortality [43]. Other studies looking at the impact of anticoagulation on survival such as Tremblay et al. did not find any improvements in overall survival of those therapeutically anticoagulated [44]. This continues to support current recommendations that therapeutic LMWH should not be used for primary prophylaxis of VTE events without further supporting evidence [45]. In contrast to these findings, a study by Paranjpe et al. found patients initiated on therapeutic dose anticoagulation in the hospital had improved survival from COVID-19 compared to those receiving prophylactic or none [46], although this study lacked information on patient demographics and reasons for initiating anticoagulation. Another study found that pre-existing anticoagulation was protective against the development of embolic events in COVID-19 patients [47] and this would seem logical given the finding that the thrombotic process had started prior to admission with 50% of patients being diagnosed with VTE within 24 h of hospitalisation [8]. The observational nature of these studies re-iterates the need for randomised controlled trials. The international REMAP-CAP trial assessing the impact of therapeutic anticoagulation on survival of patients with severe COVID-19 disease admitted to the ICU has been halted due to futility of treatment with an increase in major bleeding. However, this study continues to examine the effect of therapeutic anticoagulation in those defined to have moderate COVID-19 infection [48].

It could be inferred that patients taking chronic anticoagulation would be at an increased risk of developing thromboembolic events with more cancer and metastatic cancer and potential for acquired thrombophilia. With similar rates of AKI between those on chronic anticoagulation and those not anticoagulated it could be possible that therapeutic anticoagulation provides some protection against AKI development however randomised controlled trials would be needed to confirm this.

There are limitations to this single-centre observational study which include the inability to collect data on major bleeding and VTE events. With the high mortality in this study, especially in those already taking anticoagulant therapy, and low ICU admission rates this suggests that a high number of patients had limitations of care in place which may have excluded investigations for VTE. Also, if patients in this group were critically-ill, imaging studies may not have been performed. Early on in the course of COVID-19, the relevance of D-dimer was not well defined [4] and was not routine in our practice. Therefore, we do not have a complete dataset for D-dimer comparison of those anticoagulated and not. We did not include data on interventions of those hospitalised which may have had an impact on outcomes (e.g. those on the RECOVERY trial randomised to dexamethasone). It has not been possible to determine the cause of death for patients aside from that patients died whilst being treated for COVID-19.

## Conclusion

This study shows that chronic anticoagulation does not reduce the risk of developing AKI with COVID-19 or reduce the severity of AKI including requirement for RRT. These findings would suggest that AKI in COVID-19 is unlikely to be thrombotic in aetiology. COVID-19 related AKI is likely to be multifactorial and early detection and management is essential. RCTs are currently underway to assess whether there is a specific group of patients that therapeutic anticoagulation may provide benefit and findings from RCTs will be needed to conclusively determine the ineffectiveness of anticoagulation in preventing AKI. For patients on chronic anticoagulation the choice of therapy must depend on the patients’ current condition and may need to be adjusted based on development of AKI or initiation of interacting drugs.

## Supplementary Information

**Additional file 1: Supplementary Table 1.** Univariable logistic regression analysis for the development of AKI. **Supplementary Table 2.** Univariable cox regression analysis for overall survival. **Supplementary Figure 1.** Multivariable cox regression analysis for survival.

## Data Availability

Data cannot be shared publicly because of patient confidentiality but can be requested from lead author directly.

## References

[1] World Health Organisation. https://www.who.int/emergencies/diseases/novel-coronavirus-2019/events-as-they-happen. Accessed on 02/09/2020.

[2] Katz JM, Libman RB, Wang JJ (2020). Cerebrovascular complications of COVID-19. Stroke.

[3] Guo T, Fan Y, Chen M (2020). Cardiovascular implications of fatal outcomes of patients with coronavirus disease 2019 (COVID-19). JAMA Cardiol.

[4] Tang N, Li D, Wang X, Sun Z (2020). Abnormal coagulation parameters are associated with poor prognosis in patients with novel coronavirus pneumonia. J Thromb Haemost.

[5] Lippi G, Favaloro EJ (2020). D-dimer is associated with severity of coronavirus disease 2019 (COVID-19): a pooled analysis. Thromb Haemost.

[6] Klok FA, Kruip MJHA, van der Meer NJM (2020). Confirmation of the high cumulative incidence of thrombotic complications in critically ill ICU patients with COVID-19. Thromb Res.

[7] Poissy J, Goutay J, Caplan M (2020). Pulmonary embolism in COVID-19 patients: awareness of an increased prevalence. Circulation.

[8] Lodigiani C, Iapichino G, Carenzo L, Sandri MT, Barco S (2020). Venous and arterial thromboembolic complications in COVID-19 patients admitted to an academic hospital in Milan, Italy. Venous and arterial thromboembolic. Thromb Res.

[9] Helms J, Tacquard C, Severac F (2020). High risk of thrombosis in patients with severe SARS-CoV-2 infection: a multicenter prospective cohort study. Intensive Care Med.

[10] Cheng Y, Luo R, Wang K (2020). Kidney disease is associated with in-hospital death of patients with COVID-19. Kidney Int.

[11] Richardson S, Hirsch JS, Narasimhan M (2020). Presenting characteristics, comorbidities, and outcomes among 5700 patients hospitalized with COVID-19 in the New York City area. JAMA.

[12] Fominskiy EV, Scandroglio AM, Monti G (2021). Prevalence, characteristics, risk factors, and outcomes of invasively ventilated COVID-19 patients with acute kidney injury and renal replacement therapy. Blood Purif.

[13] Wilbers T, Koning M (2020). Renal replacement therapy in critically ill patients with COVID-19: a retrospective study investigating mortality, renal recovery and filter lifetime. J Crit Care.

[14] Zhou F, Yu T, Du R (2020). Clinical course and risk factors for mortality of adult inpatients with COVID-19 in Wuhan, China: a retrospective cohort study. Lancet.

[15] Hamilton P, Hanumapura P, Castelino L, Henney R, Parker K (2020). Characteristics and outcomes of hospitalised patients with acute kidney injury and COVID-19. PLoS One.

[16] Batlle D, Soler MJ, Sparks MA (2020). Acute kidney injury in COVID-19: emerging evidence of a distinct pathophysiology. JASN.

[17] Hirsch JS, Ng JH, Ross DW (2020). Acute kidney injury in patients hospitalized with COVID-19. Kidney Int.

[18] Kant S, Menez SP, Hanouneh M (2020). The COVID-19 nephrology compendium: AKI, CKD, ESKD and transplantation. BMC Nephrol.

[19] Su H, Yang M, Wan C (2020). Renal histopathological analysis of 26 postmortem findings of patients with COVID-19 in China. Kidney Int.

[20] Rapkiewicz AV, Mai X, Carsons SE (2020). Megakaryocytes and platelet-fibrin thrombi characterize multi-organ thrombosis at autopsy in COVID-19: a case series. EClinicalMedicine.

[21] Yao XH, Li TY, He ZC (2020). A pathological report of three COVID-19 cases by minimal invasive autopsies. Zhonghua Bing Li Xue Za Zhi.

[22] Page EM, Ariëns RAS (2021). Mechanisms of thrombosis and cardiovascular complications in COVID-19. Thromb Res.

[23] Loo J, Spittle DA (2021). Newnham MCOVID-19, immunothrombosis and venous thromboembolism: biological mechanisms. Thorax.

[24] Wang D, Hu B, Hu C (2020). Clinical characteristics of 138 hospitalized patients with 2019 novel coronavirus–infected pneumonia in Wuhan, China. JAMA.

[25] Nadim MK, Forni LG, Mehta RL (2020). COVID-19-associated acute kidney injury: consensus report of the 25th acute disease quality initiative (ADQI) workgroup. Nat Rev Nephrol.

[26] Golmai P, Larsen CP, DeVita MP (2020). Histopathologic and Ultrastructural findings in postmortem kidney biopsy material in 12 patients with AKI and COVID-19. JASN.

[27] Wu H, Larsen CP, Hernandez-Arroyo CF (2020). AKI and collapsing Glomerulopathy associated with COVID-19 and APOL1 high-risk genotype. JASN.

[28] Kudose S, Batal I, Santoriello D (2020). Kidney biopsy findings in patients with COVID-19. JASN.

[29] Sharma P, Uppal NN, Wanchoo R (2020). COVID-19–associated kidney injury: a case series of kidney biopsy findings. J Am Soc Nephrol.

[30] NICE (2020). NICE Covid-19 rapid guideline acute kidney injury in hospital.

[31] Selby NM, Forni LG, Laing CM (2020). Covid-19 and acute kidney injury in hospital: summary of NICE guidelines. BMJ.

[32] Russo E, Esposito P, Taramasso L (2021). As part of the GECOVID working group. Kidney disease and all-cause mortality in patients with COVID-19 hospitalized in Genoa, northern Italy. J Nephrol.

[33] Faculty of Intensive care medicine (ICM), Royal college of anaesthetists (RCoA), Association of Anaesthetists, Intensive care society, Royal college of physicians (2020). Clinical guide for the prevention, detection and management of thromboembolic disease in patients with COVID-19.

[34] www.thinkkidneys.nhs.uk. 2015. https://www.thinkkidneys.nhs.uk/aki/wp-content/uploads/sites/2/2015/06/Medicines-optimisation-toolkit-for-AKI-FINAL.pdf. Accessed 1 Dec 2020.

[35] Speed V, Patel RK, Byrne R, Roberts LN, Ayra R (2020). A perfect storm: root cause analysis of supra-therapeutic anticoagulation with vitamin K antagonists during the COVID-19 pandemic. Thromb Res.

[36] Williams H, Akor F. Management of patients currently on warfarin during Covid-19: Specialist Pharmacy Services; 2020. https://www.sps.nhs.uk/articles/management-of-patients-currently-on-warfarin-during-covid-19/

[37] MHRA. www.gov.uk. 2020. https://www.gov.uk/drug-safety-update/direct-acting-oral-anticoagulants-doacs-reminder-of-bleeding-risk-including-availability-of-reversal-agents#table-1%2D%2D-recommendations-for-use-of-doacs-in-patients-with-renal-impairment. Accessed 12 Nov 2020.

[38] Bikdeli B, Madhavan MV, Jimenez D (2020). COVID-19 and thrombotic or thromboembolic disease: Impli cations for prevention, antithrombotic therapy, and follow-up. J Am Coll Cardiol.

[39] Connors JM, Levy JH (2020). COVID-19 and its implications for thrombosis and anticoagulation. Blood.

[40] Mousavi S, Moradi M, Khorshidahmad T, Motamedi M (2015). Antiinflammatory effects of heparin and its derivatives: a systematic review. Adv Pharmacol Sci.

[41] Thachil J (2020). The versatile heparin in COVID-19. J Thromb Haemost.

[42] Li X, Xu S, Yu M (2020). Risk factors for severity and mortality in adult COVID-19 inpatients in Wuhan. J Allergy Clin Immunol.

[43] Knight SR, Ho A, Pius R (2020). Risk stratification of patients admitted to hospital with covid-19 using the ISARIC WHO clinical characterisation protocol: development and validation of the 4C mortality score. BMJ.

[44] Tremblay D, van Gerwen M, Alsen M (2020). Impact of anticoagulation prior to COVID-19 infection: a propensity score–matched cohort study. Blood.

[45] Spyropoulos AC, Levy JH, Ageno W (2020). Scientific and standardization committee communication: clinical guidance on the diagnosis, prevention and treatment of venous thromboembolism in hospitalized patients with COVID-19. JTH.

[46] Paranjpe I, Fuster V, Lala A (2020). Association of treatment dose anticoagulation with in-hospital survival among hospitalized patients with COVID-19. J Am Coll Cardiol.

[47] Lachant DJ, Lachant NA, Kouides P, Rappaport S, Prasad P, White RJ (2020). Chronic therapeutic anticoagulation is associated with decreased thrombotic complications in SARS-CoV-2 infection. JTH.

[48] REMAP-CAP. https://static1.squarespace.com/static/5cde3c7d9a69340001d79ffe/t/60457a757023b06241b1453c/1615166074587/REMAP-CAP+-+Therapeutic+Anticoagulation+-+V3+-+27+February+2021_WM.pdf. Accessed 02 Apr 2021.

