# Supplementary material for: Chronic anticoagulation is not associated with a reduced risk of acute kidney injury in hospitalised Covid-19 patients
Source: BMC Nephrol. 2021 Jun 16;22:224. doi: 10.1186/s12882-021-02436-5 (PMC8208381; doi:10.1186/s12882-021-02436-5)
Supplement: Supplementary file 1 — Additional file 1: Supplementary Table 1. Univariable logistic regression analysis for the development of AKI. Supplementary Table 2. Univariable cox regression analysis for overall survival. Supplementary Figure 1. Multivariable cox regression analysis for survival. [file 12882_2021_2436_MOESM1_ESM.docx]

**Supplementary Information**

Supplementary Table 1– Univariable logistic regression analysis for the development of AKI

Supplementary Table 2 – Univariable cox regression analysis for overall survival

Supplementary Figure1- Multivariable cox regression analysis for survival

| Dependent: AKI |  | OR (univariable) |
| --- | --- | --- |
| Age, years |  | 1.00 (0.99-1.01, p=0.439) |
| Sex | M | 1.45 (1.07-1.99, p=0.018) |
| Ethnicity | Asian | 0.93 (0.55-1.52, p=0.785) |
|  | Black | 2.07 (1.29-3.29, p=0.002) |
|  | Mixed | 0.70 (0.11-2.61, p=0.647) |
|  | Other | 0.32 (0.05-1.10, p=0.128) |
|  | Unknown | 1.48 (0.76-2.73, p=0.231) |
| Anticoagulation |  | 1.27 (0.85-1.88, p=0.235) |
| Hypertension |  | 1.37 (1.01-1.85, p=0.043) |
| CCI score |  | 1.13 (1.00-1.28, p=0.047) |
| Congestive Heart Failure |  | 0.92 (0.55-1.46, p=0.726) |
| Peripheral Vascular Disease |  | 1.75 (0.97-3.05, p=0.055) |
| Diabetes without complications |  | 0.97 (0.66-1.40, p=0.861) |
| Diabetes with complications |  | 3.25 (1.61-6.39, p=0.001) |
| Cancer |  | 1.73 (0.98-2.93, p=0.049) |
| Metastatic cancer |  | 1.44 (0.52-3.49, p=0.446) |
| Renal disease |  | 2.36 (1.58-3.50, p<0.001) |
| Day zero | Sodium mmol/L | 1.04 (1.02-1.06, p<0.001) |
|  | Urea mmol/L | 1.16 (1.13-1.19, p<0.001) |
|  | Creatinine | 1.01 (1.01-1.01, p<0.001) |
|  | Haemoglobin | 0.99 (0.99-1.00, p=0.090) |
|  | Lymphocytes | 1.02 (0.88-1.16, p=0.719) |
|  | CRP | 1.01 (1.00-1.01, p<0.001) |
| EWS |  | 1.18 (1.09-1.28, p<0.001) |
| Temperature | Celsius | 0.78 (0.62-0.97, p=0.030) |
| Blood pressure | Systolic mmHg | 1.00 (0.99-1.00, p=0.317) |
|  | Diastolic mmHg | 1.00 (0.98-1.01, p=0.488) |
| MAP | mmHg | 0.99 (0.98-1.01, p=0.347) |
| Oxygen saturations | Percentage | 0.94 (0.90-0.98, p=0.005) |
| Respiratory rate | Breaths/min | 1.04 (1.02-1.07, p=0.002) |

Supplementary Table 1– Univariable logistic regression analysis for the development of AKI

AKI- Acute kidney injury, CCI score – Charlson comorbidity index score, CRP- C reactive protein, EWS- Early warning score, MAP- Mean arterial pressure

|  |  | HR | 95% CI | p-value |
| --- | --- | --- | --- | --- |
| Anticoagulation |  | 1.27 | 0.97-1.67 | 0.081 |
| AKI |  | 1.48 | 1.18-1.87 | 0.001 |
| Age | *Male* | 1.03 | 1.02-1.04 | <0.001 |
| Sex |  | 1.18 | 0.94-1.47 | 0.150 |
| Ethnicity | *White* | Reference | | |
|  | *Asian* | 0.59 | 0.38-0.91 | 0.018 |
|  | *Black* | 1.13 | 0.79-1.63 | 0.502 |
|  | *Mixed* | 0.76 | 0.28-2.04 | 0.581 |
|  | *Other* | 0.67 | 0.28-1.63 | 0.379 |
|  | *Unknown* | 0.86 | 0.53-1.38 | 0.528 |
| CCI Score |  | 1.27 | 1.17-1.37 | <0.001 |
| Hypertension |  | 1.44 | 1.15-1.80 | 0.001 |
| Myocardial Infarction |  | 1.44 | 1.01-2.06 | 0.044 |
| Congestive Heart Failure |  | 2.32 | 1.78-3.02 | <0.001 |
| Peripheral Vascular Disease |  | 1.06 | 0.72-1.58 | 0.757 |
| Cerebrovascular disease |  | 1.18 | 0.75-1.83 | 0.475 |
| Dementia |  | 1.43 | 1.11-1.85 | 0.006 |
| COPD |  | 1.05 | 0.83-1.34 | 0.670 |
| Rheumatoid Disease |  | 1.21 | 0.63-2.36 | 0.566 |
| Peptic Ulcer Disease |  | 0.27 | 0.04-1.95 | 0.196 |
| Mild Liver Disease |  | 0.54 | 0.22-1.30 | 0.168 |
| Diabetes without complications |  | 1.32 | 1.04-1.68 | 0.023 |
| Diabetes with complications |  | 1.68 | 1.04-2.70 | 0.033 |
| Hemi or Paraplegia |  | 0.54 | 0.20-1.44 | 0.215 |
| Renal Disease |  | 1.56 | 1.20-2.02 | 0.001 |
| Moderate/Severe Liver Disease |  | 1.73 | 0.71-4.19 | 0.224 |
| Cancer |  | 1.94 | 1.39-2.70 | <0.001 |
| Metastatic cancer |  | 2.23 | 1.28-3.89 | 0.005 |
| AIDS |  | 5.62 | 0.79-40.20 | 0.085 |

Supplementary Table 2 – Univariable cox regression analysis for overall survival

AKI- Acute kidney injury, CCI score – Charlson comorbidity index score, COPD – chronic obstructive pulmonary disease, AIDS- acquired immunodeficiency syndrome

Supplementary Figure1- Multivariable cox regression analysis for survival

AKI- Acute kidney injury, CCI score – Charlson comorbidity index score, COPD – chronic obstructive pulmonary disease, AIDS- acquired immunodeficiency syndrome
